# Association between dietary anthocyanidins intake and depression among US adults: a cross-sectional study (NHANES, 2007–2010 and 2017–2018)

**DOI:** 10.1186/s12888-023-05029-8

**Published:** 2023-07-20

**Authors:** Wen-li Chen, Jing Zhao

**Affiliations:** Department of Ophthalmology, Zhejiang Chinese Medical University Affiliated Wenzhou Hospital of Integrated Traditional Chinese and Western Medicine, Wenzhou, 325000 Zhejiang China

**Keywords:** Anthocyanidins, Depression, NHANES, Multiple logistic regression

## Abstract

**Background:**

Anthocyanidins encompass a diverse array of compounds that possess notable anti-inflammatory and antioxidant properties with pharmacological activity. However, the correlation between the consumption of anthocyanidins through diet and its impact on depression has yet to be investigated.

**Methods:**

This study utilized the Food and Nutrient Database for Dietary Studies (FNDDS) expanded flavonoid intake database, as well as data from the National Health and Nutrition Examination Survey (NHANES) from the years 2007 to 2010 and 2017 to 2018. The analysis of the collected data was conducted in R, following the guidelines outlined in the official NHANES user guide “Stratified Multi-stage Probability Sampling”. Three different models were developed using logistic regression to assess the protective effects of T3 (representing the highest intake of anthocyanidins) against depression. Additionally, the study aimed to investigate whether there existed a non-linear relationship between the dietary intake of anthocyanidins and the prevalence of depression by employing restricted cubic spline (RCS) analysis.

**Results:**

A total of 6,845 eligible participants were included in this cross-sectional study, with their data appropriately weighted to represent a population of 89.8 million people in the United States of America. The results demonstrated that individuals diagnosed with depression had a significantly lower dietary intake of anthocyanidins compared to those without depression (*P *< 0.0001). Moreover, significant differences were observed among different participant groups regarding socioeconomic status and the presence of chronic physical illnesses (such as hypertension, glucose status, and chronic kidney disease risk, etc.) (*P *< 0.05). After adjustment for covariates, participants with the highest intake of anthocyanins (T3) demonstrated a significantly reduced risk of depression [OR_T3_ = 0.67, 95%CI: (0.48–0.95), (*P*_trend_= 0.02]. Furthermore, the RCS analysis revealed a significant linear relationship between dietary anthocyanidin intake and depression (*P* for non-linear = 0.5876).

**Conclusion:**

Our findings reveal a negative association between dietary anthocyanidin intake and depression.

## Introduction

Depression is a global public health concern and is ranked as the second most serious health problem worldwide, following cardiovascular disease. It is considered a leading cause of disability and death across different populations [1]. Epidemiological studies estimate that approximately 322 million people worldwide are affected by depression, imposing a significant social and economic burden. The global prevalence of major depressive disorder has also seen a significant increase of 27.6% in 2020 due to the COVID-19 pandemic [2, 3]. Predictive models suggest that depression will become the leading cause of the global burden of disease by 2030 [4]. Depression not only impacts an individual’s emotional well-being but also leads to a decline in muscle strength and motor function [5, 6]. Therefore, it is crucial to identify actionable factors that influence and alleviate depression in the population. Although the exact pathogenesis of depression is not yet fully understood, it is undeniable that dietary differences among individuals play a significant role in the occurrence and development of depression [7, 8]. Consequently, further in-depth exploration of potential dietary strategies for preventing and managing depression is urgently needed.

There is mounting research evidence linking dietary factors, particularly nutrients, to the risk of depression. For instance, an elevated index of dietary inflammation has been identified as a risk factor for depression [9]. In addition, a low-dose vitamin K diet has been shown to inhibit the activity of hippocampal neurons, potentially increasing oxidative stress levels in the brain and the risk of depression [10]. Previous studies have also demonstrated a strong association between other dietary micronutrients such as magnesium, calcium and fruits and a higher risk of depression [11–13]. However, there is limited research investigating the relationship between dietary flavonoid intake and depressive symptoms.

Numerous in vitro and animal model studies have highlighted the potential role of flavonoids in improving depressive states. Pharmacological research has shown that isoliquiritin can reduce the NLRP3-mediated inflammatory pyroptosis pathway by regulating the miRNA-27a/SYK/NF-κB axis, thereby improving the depressive state in mice [14]. Flavonoids possess various biological activities beneficial to human health, and their main mechanism of action involves modulating the type and structure of intestinal microorganisms and enhancing brain neuron activity through the gut-brain signaling pathway axis, consequently reducing depressive symptoms [15]. While these findings suggest that flavonoids may have therapeutic effects on depression, clinical evidence linking flavonoid intake, including specific subclasses, to the risk of depression is currently lacking. Hence, based on the Food and Nutrient Database for Dietary Studies (FNDDS) and NHANES, we conducted a cross-sectional study to investigate the relationship between dietary flavonoid intake and the risk of depression.

## Methods

### Study design and participants

**Fig. 1:**
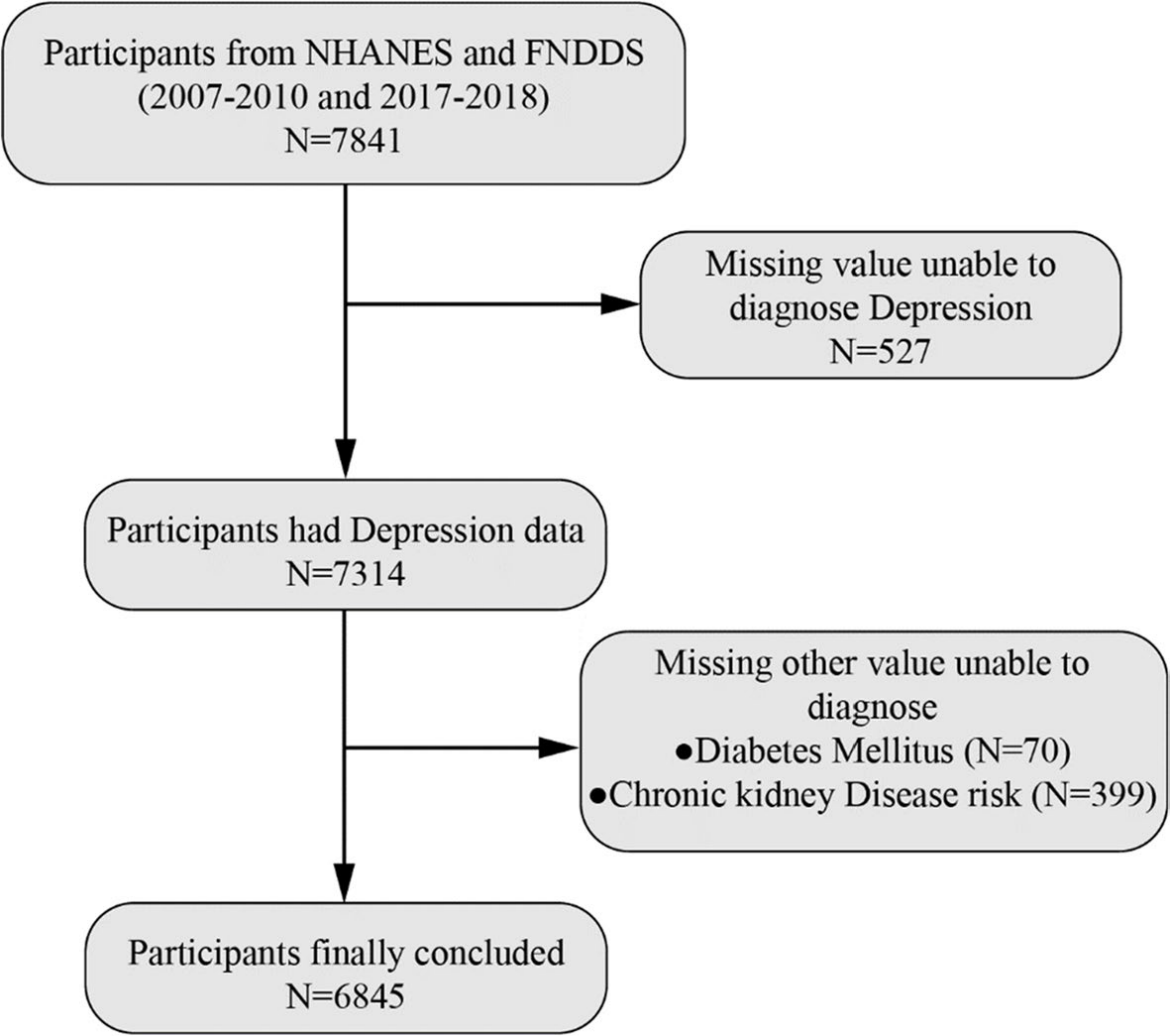
A flowchart of participant selection. NHANES, National Health and Nutrition Examination Survey; FNDDS, Food and Nutrient Database for Dietary Studies

We employed three cycles of relevant datasets from NHANES (2007–2010 and 2017– 2018), which is a national cross-sectional population-based survey. These specific cycles were chosen because NHANES collected data on dietary intake of flavonoids through a 24-hour dietary recall during this time period. NHANES uses a complex multi-stage probabilistic design to assess the physical health status of the entire US population by recruiting a representative sample from US resident communities. The comprehensive study design and data collection methods have been previously described [16]. The survey conducted for this study received approval from the US National Committee for Ethical Review of Health Statistics Research, and all participants provided written informed consent. Considering that FNDDS only releases the dietary intake data of flavonoids for 3 cycles (2007–2009, 2009–2010 and 2017– 2018), the inclusion and exclusion of the population only considers the year data of the same cycle. This study primarily included individuals aged 18 years and older, while data without a diagnosis of depression and missing dietary information on flavonoids were excluded (Fig. 1).

### Assessment of dietary intake of flavonoids and anthocyanins

Flavonoid intake in this study was based on the consumption of food and beverages, including water, and did not take into account the intake of flavonoid supplements or medications. The quantification of flavonoid intake was standardized according to the United States Department of Agriculture Expanded Flavonoid Database. NHANES provided sample weights specifically designed for dietary analysis. the intake of different flavonoids was calculated by multiplying the flavonoid content of each food by the food-frequency questionnaire (FFQ) [17]. The 24-hour recalled dietary intake type was obtained through the FFQ questionnaire, and then the different types of things were coded according to the FNDDS manual, and the flavonoid content of the things represented by different codes was calculated. Qualified dietitians collected dietary data, including flavonoid intake and other nutrients, through 24-hour food recall interviews. The FNDDS versions used for different survey cycles were version 4.1 for 2007–2008 and version 5.0 for 2009–2010 and 2017–2018 [18]. The FNDDS categorizes dietary flavonoids into six subgroups: Anthocyanidins, Flavan-3-ols, Flavanones, Flavones, Flavonols, and Isoflavones. Additionally, it estimates the total daily intake of all flavonoids, which is the cumulative sum of the 29 individual flavonoids considered.

### Evaluation of depressive symptoms

In line with a previously published NHANES-based article, the screening of depressive symptoms in this study was conducted using the Patient Health Questionnaire-9 (PHQ-9) program and computer-assisted personal interviews at mobile screening centers [19]. The PHQ-9 is a reliable and effective screening tool consisting of nine items that assess about the frequency of depressive symptoms in the past two weeks. Each item provides response options ranging from “not at all” to “almost every day”, with and the scores assigned from 0 to 3 points accordingly. The total score on the PHQ-9 ranges from 0 to 27 points, with higher scores indicating more severe depressive symptoms. A PHQ-9 total score exceeding10 is typically considered indicative of depressive symptoms, while scores below 10 indicate a non-depressed state [20].

### Study covariates

The covariates included in this study were as follows: Among them, age, body mass index (BMI), poverty, Homeostatic Model Assessment for Insulin Resistance (HOMA_IR), energy intake (Kcal), Healthy Eating Index (HEI2015), waistline as continuous variables, while sex (female or male), education level (less than 9th grade- including uneducated participants, 9-11th grade, high school graduate/GED or equivalent, some college or AA degree, college graduate or above), race (Black, Mexican American, White, Other Hispanic and Other Race), glucose status, hypertension, alcohol or smoke use, chronic kidney disease (CKD) risk and physical activity are included as categorical variables.

### Statistical analysis

To ensure the representative nature of the sample within the overall population, the analysis was weighted using the appropriate sample weights provided by the official NHANES website. The complex multistage cluster survey design of NHANES was taken into account. Continuous variables were presented as mean (standard deviation, SD), while categorical variables were expressed as number (percentage, %). respectively. Differences in baseline characteristics between the normal and glaucoma groups were compared using the weighted Student’s t-test for continuous variables) and the weighted chi-squared test for categorical variables. Flavonoid intakes were adjusted for total energy intake by using the residual method [21].

Three models were constructed for the analysis: Model 1: This was the raw model without any adjustments for covariates. Model 2: Adjustments were made for race/ethnicity(eth), poverty, sex, and age, in addition to the covariates. Model 3: In addition to the covariates adjusted in Model 2, further adjustments were made for HOMA_IR, hypertension, BMI, physical activity, energy intake, glucose status and waistline. The restricted cubic spline (RCS) function, a powerful tool, was utilized to describe the dose-response relationships between continuous variables and outcomes. All analyses were performed using the “nhanesR” package in R version 4.2.3. A significance level of *P *< 0.05 was considered statistically significant.

## Results

### Characteristics of the study participants

A total of 6,845 participants from NHANES (2007–2010 and 2017–2018) were included in the study following the established screening protocol, representing approximately 89.8 million inhabitants of the United States. Among these participants, 637 individuals (9.31%) had a diagnosis of depression (Fig. 1; Table 1).

Table 1, provides an overview of the demographic and baseline clinical characteristics of the participants. Among all the participants, 3,461 (50.56%) were female, and non-Hispanic whites constituted the largest proportion, accounting for over 40% of the population (3,053, 44.6%). The mean age of all participants was 50.15 years, with a standard deviation of 17.75 years. Notably, significant differences were observed in demographic and baseline clinical characteristics between participants diagnosed with depression and those without depression. Participants with depression tended to have lower educational attainment [240 (42.12%) vs. 3137 (58.49%)], higher levels of poverty [1.79 (1.43) vs. 2.60 (1.61)], higher BMI [30.62 (7.88) vs. 29.06 (6.68)], higher HOMA_IR [5.90 (11.42) vs. 4.01 (6.11)], lower HEI-2015 scores [46.70 (13.16) vs. 50.80 (13.75)] and higher waistline measurements [102.19 (17.66) vs. 99.19 (16.03)]. Additionally, smoking status appeared to influence the occurrence of depression. Furthermore, participants with poor glucose status and inadequate hypertension control were more likely to be diagnosed with depression. All of the aforementioned differences between the depression and non-depression groups were statistically significant (*P* < 0.05).

**Table 1:** Characteristics of the study population, weighted

Variable	Total (n = 6845)	Depression		*P* value
	**Yes(n = 637)**	**No(n = 6208)**		
Age (mean, SD)	50.15 ± 17.75	49.36 ± 15.61	50.23 ± 17.91	0.189
Sex (n, %)				**< 0.0001**
Female	3461(50.56)	420(65.89)	3041(49.91)	
Male	3384(49.44)	217(34.11)	3167(50.09)	
Race (n, %)				**0.004**
Black	1325(19.36)	142(16.05)	1183(10.40)	
Mexican American	1174(17.15)	107(8.83)	1067(8.80)	
White	3053(44.6)	264(58.90)	2789(68.35)	
Other Hispanic	727(10.62)	91(8.57)	636(5.46)	
Other Race	566(8.27)	33(7.66)	533(6.99)	
Education (n, %)				**< 0.0001**
Less than 9th grade	838(12.24)	110(9.46)	728(6.01)	
9-11th grade (including 12th grade with no diploma)	1016(14.84)	139(18.93)	877(10.00)	
High school graduate/GED or equivalent	1614(23.58)	148(29.48)	1466(25.50)	
Some college or AA degree	1933(28.24)	184(30.63)	1749(29.65)	
College graduate or above	1444(21.1)	56(11.49)	1388(28.84)	
Poverty (mean, SD)	2.53 ± 1.61	1.79 ± 1.43	2.60 ± 1.61	**< 0.0001**
BMI (mean, SD)	29.21 ± 6.82	30.62 ± 7.88	29.06 ± 6.68	**< 0.0001**
HOMA_IR (mean, SD)	4.19 ± 6.80	5.90 ± 11.42	4.01 ± 6.11	**< 0.0001**
Kcal (mean, SD)	2078.24 ± 982.10	1969.48 ± 1030.25	2089.40 ± 976.43	0.003
HEI2015 (mean, SD)	50.42 ± 13.75	46.70 ± 13.16	50.80 ± 13.75	**< 0.0001**
Waistline (mean, SD)	99.46 ± 16.20	102.19 ± 17.66	99.19 ± 16.03	**< 0.0001**
Physical activity (n, %)				**< 0.0001**
Inactive	3700(54.05)	461(72.37)	3239(52.17)	
Active	3145(45.95)	176(27.63)	2969(47.83)	
Smoke (n, %)				**< 0.0001**
former	1728(25.24)	139(20.82)	1589(26.56)	
never	3729(54.48)	262(37.86)	3467(55.92)	
now	1388(20.28)	236(41.32)	1152(17.53)	
Alcohol (n, %)				0.07
never	888(12.97)	95(10.77)	793( 9.92)	
former	971(14.19)	106(12.50)	865(10.85)	
mild	2497(36.48)	168(32.16)	2329(40.88)	
moderate	1093(15.97)	104(17.45)	989(17.29)	
heavy	1396(20.39)	164(27.11)	1232(21.06)	
Hypertension (n, %)				**0.002**
no	3894(56.89)	308(51.45)	3586(63.23)	
yes	2951(43.11)	329(48.55)	2622(36.77)	
Glucose status (n, %)				**0.02**
DM	1512(22.09)	191(22.76)	1321(15.67)	
IFG	763(11.15)	57(11.75)	706(11.48)	
IGT	444(6.49)	37(5.75)	407(6.16)	
no	4126(60.28)	352(59.75)	3774(66.69)	
CKD risk (n, %)				0.76
Low risk	5633(82.29)	516(84.84)	5117(86.47)	
Moderate risk	815(11.91)	81(10.86)	734( 9.67)	
High risk	238(3.48)	25(2.60)	213(2.33)	
Very high risk	159(2.32)	15(1.69)	144(1.52)	

*DM*  Diabetes mellitus, *IFG* Impaired fasting glucose, *IGT* Impaired glucose tolerance, *HOMA_IR* Homeostatic Model Assessment for Insulin Resistance, *BMI* Body mass index, *CKD* Chronic kidney disease risk

### Relationship between dietary anthocyanidin intake and depression

We analyzed the relationship between the major categories of flavonoids and total intake, specifically focusing on anthocyanidins. Our findings revealed that individuals without depression had a higher intake of anthocyanidins compared to those diagnosed with depression [13.53 (0.87) mg vs. 5.81 (1.01) mg; *P *< 0.0001]. Conversely, the intake of the other five flavonoid categories did not show any statistically significant differences. Further analysis of the subclasses of anthocyanidins demonstrated that all of them (cyanidin, petunidin, delphinidin, malvidin, pelargonidin and peonidin) exhibited the same trend (Table 2).

**Table 2:** Differences in flavonoid content between the depression and non-depression groups

Variable	Total		Diagnosed with depression	No depression	*P* value
Total isoflavones, mg/d		1.64 ± 11.10	1.03 ± 5.35	1.70 ± 11.52	**0.15**
Total anthocyanidins, mg/d		11.38 ± 33.91	6.14 ± 18.23	11.92 ± 35.08	**< 0.0001**
Total flavan-3-ols, mg/d		155.08 ± 398.90	176.05 ± 446.04	152.93 ± 393.72	0.21
Total flavanones, mg/d		12.92 ± 31.53	13.05 ± 33.25	12.91 ± 31.35	0.92
Total flavonols, mg/d		0.85 ± 1.33	0.88 ± 1.35	0.63 ± 0.11	0.55
Total flavones, mg/d		17.78 ± 19.13	17.78 ± 20.63	17.78 ± 18.97	1.00
Total sum of all flavonoids, mg/d		199.63 ± 417.37	214.66 ± 463.96	198.09 ± 412.30	0.38
Subclasses of anthocyanidins					
Cyanidin, mg/d	2.23 ± 10.69		1.66 ± 7.67	2.29 ± 10.95	0.16
Petunidin, mg/d	0.88 ± 10.10		0.39 ± 1.82	0.93 ± 4.26	**< 0.0001**
Delphinidin, mg/d	1.27 ± 5.93		0.56 ± 2.44	1.34 ± 6.17	**< 0.0001**
Malvidin, mg/d	4.04 ± 15.78		2.18 ± 9.14	4.23 ± 16.29	**< 0.0001**
Pelargonidin, mg/d	1.43 ± 6.83		0.64 ± 3.01	1.51 ± 7.10	**< 0.0001**
Peonidin, mg/d	1.54 ± 10.31		0.71 ± 10.95	1.62 ± 10.70	**0.03**

The consumption of all variables was calculated as energy-adjusted values of flavonoid intake using a residual approach

### Higher intake of anthocyanidins is associated with lower risk of depression

Table 3 presents the results of the multiple logistic regression model after adjusting for different covariates. The analysis revealed that individuals in the highest tertile (T3) of anthocyanidin intake had a reduced risk of being diagnosed with depression compared to those in the lowest tertile (T1), as observed in Model 1 [OR_T3_ = 0.49, 95%CI: (0.37–0.66), *P*_trend_ < 0.0001]. After adjustment for covariates, significant differences were still observed between the highest tertile (T3) of anthocyanidin intake and the lowest tertile (T1) in both Model 2 [OR_T3_ = 0.57, 95%CI: (0.42–0.78), *P*_trend_ <0.001] and Model 3 [OR_T3_ = 0.67, 95%CI: (0.48–0.95), *P*_trend_  = 0.02]. These results indicated that a higher intake of anthocyanidins was associated with a reduced risk of clinically diagnosed depression.

**Table 3:** Multivariate logistic regression analysis of the association between anthocyanidins intake with the risk of depression, weighted

Variable	Tertile 1(Low)	Tertile 2(Moderate)	Tertile 3(High)	*P* for trend
Anthocyanidins				
Median (range, mg/d)	0.00[0.00,0.00]	0.78(0.00,3.27]	14.79(3.27,870.48]	
Model 1 [OR (95% CI)]	Reference	0.93(0.72,1.20)	**0.49(0.37,0.66)**	**< 0.0001**
Model 2 [OR (95% CI)]	Reference	0.91(0.68,1.23)	**0.57(0.42,0.78)**	**< 0.001**
Model 3 [OR (95% CI)]	Reference	0.95(0.67,1.33)	**0.67(0.48,0.95)**	**0.02**

Adjusted multinomial logistic regression of anthocyanidins intake with the risk of depression

Model 1: No covariates were adjusted; Model 2: eth, poverty, sex and age were adjusted; Model 3: eth, poverty, sex, age, *HOMA_IR* Hypertension, *BMI* Physical activity, energy intake, glucose status and waistline were adjusted

*P*<0.05 indicates a significant difference. *OR* Odds ratio, *95% CI* 95% confidence interval

### Nonlinear relationship between intake of anthocyanidins and the risk of depression

**Fig. 2:**
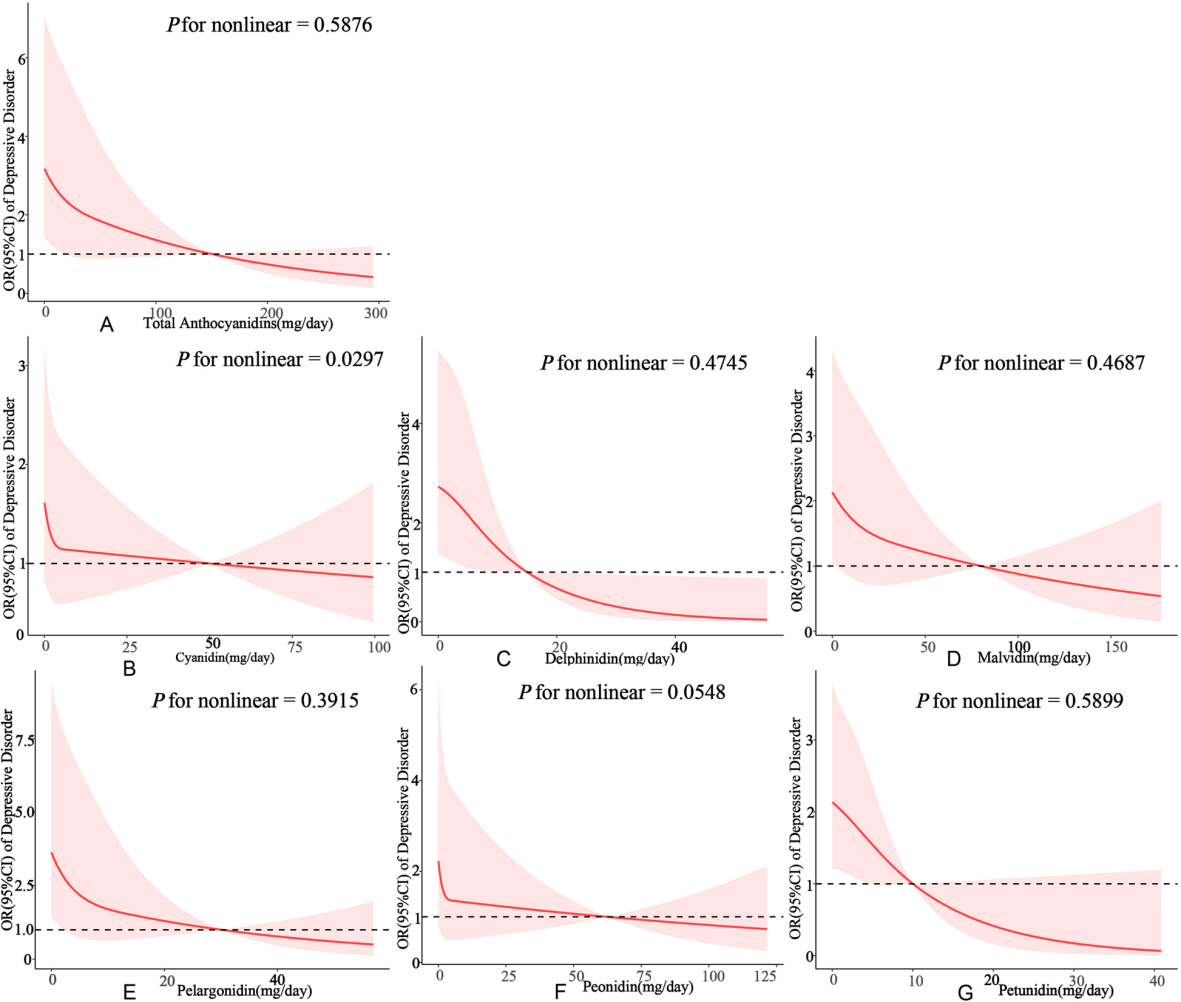
The non-linear relationships between anthocyanidin intake and the risk of depression by restricted cubic splines (**A**: Total anthocyanidins; **B**: Cyanidin; **C**: Delphinidin; **D**: Malvidin; **E**: Pelargonidin; **F**: Peonidin; **G**: Petunidin). Models are adjusted for eth, poverty, sex, age, HOMA_IR, hypertension, BMI, physical activity, energy intake, glucose status and waistline

RCS curves were used to determine potential nonlinearity in the relationship between anthocyanidin intake and the risk of depression (Fig. 2). The curves displayed a nonlinear pattern for different anthocyanidins. However, the statistical analysis indicated that there was no significant evidence of nonlinearity for Total Anthocyanidins (*P* for nonlinearity = 0.5876), Delphinidin (*P* for nonlinearity = 0.4745), Malvidin (*P* for nonlinearity = 0.4687), Pelargonidin (*P* for nonlinearity = 0.3915), Petunidin (*P* for nonlinearity = 0.5899) and Peonidin (*P* for nonlinearity = 0.0548). These anthocyanidins were all found to have a significantly negative and linear association with the risk of depression. Furthermore, a significant J-shaped association was observed between the intake of cyanidin and peonidin and the risk of depression (*P* for nonlinearity = 0.0297). In summary, the overall trend indicated that the risk of depression tended to decrease with increasing anthocyanidin intake. Overall, the risk of depression tended to decrease with increasing anthocyanidin intake.

## Discussion

While the association between dietary habits and the prevalence of depression has been established, the specific markers of diet that predict the risk of depression are still unclear. To our knowledge, this study is the first to analyze the relationship between dietary flavonoid intake and the risk of depression. We were surprised to discover that dietary anthocyanin intake, when examined categorically, showed an inverse association with depression in both the 2007–2010 and 2017–2018 NHANES datasets. After categorizing the continuous variables, we observed a 40% reduction in the incidence of depression when individuals in the highest intake group (T3) consumed dietary anthocyanidins, even after adjusting for potential confounding factors. Furthermore, the RCS curves demonstrated a linear negative correlation between anthocyanidin intake and the risk of diagnosed depression, indicating that anthocyanidin intake can serve as a reliable predictor of depression in the adult population.

Anthocyanidins are polyphenolic phytochemicals predominantly found in fruits, seeds, flowers, and green vegetables. Previous research has established that eating a regular, low-carb diet can reduce an individual’s risk of developing symptoms of stress [22, 23]. These compounds which have recently been reported to exert beneficial effects on mood and mood disorders. Moreover, emerging studies suggest that neurotransmitter imbalances, inflammatory responses (i.e., cytokines), and oxidative stress (i.e., reactive oxygen species and lipid peroxidation) may underlie the progression of depression [24, 25]. Clinical evidence has demonstrated significantly higher concentrations of inflammatory cytokines, such as IL-1β, IL-6 and TNF-α activated by NLRP3 in the cerebrospinal fluid and serum of patients with depression compared to non-depressed individuals [26]. Due to their unique chemical structures, anthocyanidins possess various pharmacological activities, including antioxidant, anti-inflammatory, antidepressant and neuroprotective, properties. This makes them potential candidates for future antidepressant medications.

Epidemiological studies investigating the association between anthocyanin intake and depression have been limited. A double-blind, randomized, placebo-controlled, parallel-group study showed that an 8-week intake of Purple-Flesh Potato, which is rich in anthocyanins, resulted in improved psychological stress response, reduced irritability, and alleviation of depressive symptoms [27]. Another randomized, double-blind, placebo-controlled clinical trial focusing on adolescents who consumed a daily supplement of wild blueberry (containing approximately 253 mg anthocyanidins) found that compared to the placebo group, participants receiving anthocyanidin supplements had significantly reduced self-reported depressive symptoms (*P* = 0.02, 95%CI: (-6.71,-5.35)] [28]. Previous cross-sectional studies have also demonstrated that correlation between higher diet quality, as indicated by higher scores on the Healthy Eating Index HEI2015), and a lower risk of depression [29–31]. Our study further confirms this association [46.09 (0.66) vs. 50.85 (0.47), *P<*0.0001]. These findings suggest that consuming an adequate amount of anthocyanins may have a modestly effective potential intervention for preventing depression. While there is strong evidence supporting the protective effects of dietary anthocyanins against oxidative stress and neurological damage, our study is the first to establish reveal a link between dietary anthocyanin intake and the presence of depression in the general population.

As described earlier, differences in dietary anthocyanidin intake have been strongly associated with the risk of depression, although the underlying mechanisms have not been fully elucidated. Based on our literature search, we have summarized several potential pathological mechanisms. These mechanisms include the reduction of oxidative stress and inflammation, as well as changes in the gut microbiota, which are involved in chronic affective-cognitive disorders. Firstly, the imbalance between the production and clearance of reactive oxygen species (ROS), known as represented by oxidative stress, is believed to play a crucial role in the development and progression of depression. Excessive ROS can attack cells, leading to protein and lipid peroxidation, damage to the DNA molecular structure, and ultimately cell death [32]. Anthocyanidins, as natural antioxidants, have the ability to neutralize various types of reactive oxygen species, including superoxide anions, singlet oxygen and peroxide free radicals [33]. Secondly, chronic neuroinflammation is known to play a significant role in the progression of depression [34]. Anthocyanidins have been shown to improve the brain microenvironment by inhibiting the activity of nuclear factor-kappa B (NF-κB) and reducing inflammation levels [35]. Lastly, adequate intake of anthocyanidins can positively influence the composition of the gut microbiota, which in turn can modulate the bidirectional communication between the microbiota and the brain, known as the microbiota-gut-brain axis [36]. This can lead to an improvement in the nerve conduction excitability of the brain. At the same time, phenolic metabolites represented by Malvidin have beneficial effects on anxiety and depression by targeting and regulating NLRP3, NLRC4 and AIM2 inflammasomes [37]. Although existing evidence suggests that sufficient intake of anthocyanins is beneficial for individuals with depression, further research is needed to better understand the underlying mechanisms. Prospective clinical studies with larger sample sizes and mechanistic investigations are necessary to clarify the relationship between dietary anthocyanidin intake and depression.

This study has some limitations that should be acknowledged. Firstly, the differences in demographics and population characteristics in the United States may limit the generalizability of the findings to other countries or regions. Secondly, the assessment of depressive symptoms relied on self-reported measures, and dietary assessments using 24-hour dietary recall interviews may introduce errors in estimating actual intake. The quantification of cyanine intake also lacks objective and quantitative indicators. Finally, this study is cross-sectional in nature, which limits the ability to establish causal relationships, especially when compared to longitudinal or clinical randomized controlled trials.

## Conclusion

In conclusion, our findings provide the first evidence of an inverse association between dietary anthocyanin intake and the risk of depressive symptoms in adults in the United States. The prevalence of depressive symptoms decreased as anthocyanin intake increased. Nevertheless further prospective studies are warranted to examine the potential role of other factors such as calcium in relation to depressive symptoms.

## Data Availability

Data generated and analyzed in the current study were sourced from the NHANES website (https://wwwn.cdc.gov/nchs/nhanes/analyticguidelines.aspx) and the FNDDS website (https://www.ars.usda.gov/northeast-area/beltsville-md-bhnrc/beltsville-human-nutrition-research-center/food-surveys-research-group/docs/fndds-flavonoid-database/), data release cycle: 2007–2010 and 2017–2018.
